# Easy Handling of Sensors and Actuators over TCP/IP Networks by Open Source Hardware/Software

**DOI:** 10.3390/s17010094

**Published:** 2017-01-05

**Authors:** Andrés Mejías, Reyes S. Herrera, Marco A. Márquez, Antonio José Calderón, Isaías González, José Manuel Andújar

**Affiliations:** 1 Department of Electronic, Computer Science and Automation Engineering, University of Huelva, Escuela Técnica Superior de Ingeniería, Ctra. Huelva-Palos de la Fra, 21819 Huelva, Spain; andujar@diesia.uhu.es; 2 Department of Electrical Engineering, University of Huelva, Escuela Técnica Superior de Ingeniería, Ctra. Huelva-Palos de la Fra, 21819 Huelva, Spain; reyes.sanchez@die.uhu.es; 3 Control and Robotics Research Group, University of Huelva, Escuela Técnica Superior de Ingeniería, Ctra. Huelva-Palos de la Fra, 21819 Huelva, Spain; marcoa@pi.uhu.es; 4 Department of Electrical Engineering, Electronics and Automation, University of Extremadura, Avenida de Elvas, s/n, 06006 Badajoz, Spain; ajcalde@unex.es (A.J.C.); igonzp@unex.es (I.G.)

**Keywords:** Modbus, remote access, open source hardware/software, collaborative access, EJS, Arduino, data acquisition, Internet-of-Things, HMI

## Abstract

There are several specific solutions for accessing sensors and actuators present in any process or system through a TCP/IP network, either local or a wide area type like the Internet. The usage of sensors and actuators of different nature and diverse interfaces (SPI, I2C, analogue, etc.) makes access to them from a network in a homogeneous and secure way more complex. A framework, including both software and hardware resources, is necessary to simplify and unify networked access to these devices. In this paper, a set of open-source software tools, specifically designed to cover the different issues concerning the access to sensors and actuators, and two proposed low-cost hardware architectures to operate with the abovementioned software tools are presented. They allow integrated and easy access to local or remote sensors and actuators. The software tools, integrated in the free authoring tool Easy Java and Javascript Simulations (EJS) solve the interaction issues between the subsystem that integrates sensors and actuators into the network, called *convergence subsystem* in this paper, and the Human Machine Interface (HMI)—this one designed using the intuitive graphical system of EJS—located on the user’s computer. The proposed hardware architectures and software tools are described and experimental implementations with the proposed tools are presented.

## 1. Introduction

Network-based communications allow the interconnection of sensors, actuators, control units, and computers under diverse topologies, from point-to-point connection to complex networks composed by hundreds of nodes or devices. Information transmission over a network provides important benefits in terms of reliability, reconfigurability, enhanced resource utilization, reduced wiring, and easier diagnosis and maintenance. In addition, networked communication allows taking advantage of modern data platforms and cloud-hosted services [1].

Continuous improvements in Information and Communication Technologies (ICT) are increasing the connectivity in every aspect of the modern world. A new version of the Internet Protocol (version 6, IPv6) has even been specified in order to overcome the address shortage of the traditional IPv4 addressing [2]. On the other hand, advances in sensor and communication technologies can provide the foundations for linking the physical facilities to the cyber world of Internet applications and the software world [3]. As a consequence, the concept of the Internet-of-Things (IoT) has emerged and constitutes a current promising research field. IoT deals with the interconnection of all types of embedded computing devices [4]. In other words, IoT could be described as the pervasive and global network, which provides a system for the monitoring and control of the physical world through the collection, processing and analysis of generated data by IoT devices [5]. Within this context, another dawning concept is the Cyber-Physical Systems (CPS) scenario in which machines have advanced communication capabilities. In CPS various embedded devices are networked to sense, monitor and actuate physical elements in the real world [6]. In the industry, the migration to IP for monitoring and automation is becoming increasingly popular, as Transmission Control Protocol/Internet Protocol (TCP/IP) connections offer real-time monitoring and maintenance processes, peer-to-peer communication between Remote Terminal Units (RTUs), multiple sessions, concurrency and security services [7]. In fact, in the industrial domain, the IoT is expected to bring about a fourth industrial revolution based on the industry-wide adoption of CPS [8]. By means of communication modules, TCP/IP-based solutions (hard and soft) may contribute to make remotely accessible the legacy commercial equipment that does not support Internet connectivity [1]. Concerning the adoption of IoT-based systems, in recent years, there has been a shift from systems based around the interconnection of physical components in which transmitted data has been used to facilitate control, to systems in which information is at the heart of the system and serviced by smart objects [9].

The intelligent networks of power generation and distribution, called Smart Grids (SGs) constitute another modern research arena where TCP/IP networks are applied. SGs are also inside the CPS concept since they are a special example of a cyber-physical infrastructure [10].

A particular case of application of networked communications that has attracted a lot of attention during last years is engineering education. From the point of view of engineering education, small/lab scale plants, that have a similar operation in their fundamental aspects to plants or industrial systems, constitute an essential tool for students to acquire skills for their professional future [11,12]. In many cases, these educational plants contain sensors and actuators similar to those that can be found in most of the common industrial systems. Besides, they cover many fields in engineering, such as building management, electrical power, renewable energies, electronics, electrical machines, hydraulics, etc. Some examples of these products can be seen at [13,14,15].

Access to these educational plants can be carried out basically in two ways: locally and remotely. Local access assumes that the user attends in person lab classes where can program and/or configure these plants. Several limitations arise when they are used in this way: the number of plants is reduced because of their high cost, and some plants can mean dangerous to the student if they are improperly operated, such as motors and generators of some power. Time availability of educators is also an aspect to be taken into account. Conversely, remote access is emerging as one of the most important trends in the use of laboratories [16,17,18,19]. This kind of access may improve some shortcomings of local access, such as safety aspect of users and the possibility that plants can be used 24 h a day, without being limited to the university schedule. However, access to these plants constitutes a major technological challenge, due to the many aspects that need to be addressed (communications, design of user interfaces, access to different sensors, performing actions on the actuators, communications security, etc.). Various works perform in-depth reviews of virtual and remote labs in engineering education emphasizing the benefits of their use [18,20,21,22]. Particularly for control education, a recent survey is reported in [19].

Regardless of the context, IoT, CPS, etc., the handling of sensors and actuators and, hence, the associated data is performed over TCP/IP networks. This kind of communication needs special attention from the scientific community to resolve some pending challenges among which remote access [7]. Nowadays, there are several specific solutions to access different kinds of sensors and actuators through a TCP/IP network, either local (Local Area Network, LAN) or wide area type (Wide Area Network, WAN). The usage of sensors and actuators with different interfaces (SPI, I2C, analogue, digital, serial…) make the access to them from a network in a homogeneous way more complicated. If secure access to these devices is required (by encrypting communications), the design of the entire system becomes more and more complex. Taking into account these requirements, a framework, including both software and hardware resources, is necessary to simplify and unify networked access of diverse sensors and actuators. Such a framework constitutes a tool that would greatly simplify the design of management systems, monitoring interfaces, alarm systems, etc.

One of the main problems that often arise when implementing remote access to a system (usually including actions on actuators, implementation of control strategies, design of sensors monitoring systems, etc.) is the lack of software and hardware tools to approach the design of the whole scenario that is necessary to fulfill. Moreover, in many cases, only commercial software tools are available, which means an expense that is unaffordable in several applications. In the literature we can find many examples of access to remote systems where implementation is performed using commercial software, ad hoc designs or a mixture of both solutions [23,24,25,26,27,28,29,30,31,32,33]. In this regard, in the path towards the future generation of technological processes, there is a trend to replace proprietary approaches with open and standardized solutions [34]. In this sense, Botta et al. [35] assert that research efforts must be done in the direction of defining standard protocols, libraries, languages, and methodologies for enabling the full potential of IoT. Open source platforms are a viable alternative to commercial packages. The first ones offer benefits like more control over the system and data, more low-level configurability to the developer, and the collected data does not have to reside on third party databases [1].

Easy Java/Javascript Simulations (EJS) [36] is an open-source tool created by Esquembre [37] to build discrete simulations. Based on Java, this software provides an environment for rapid development of interactive virtual laboratories. In addition, EJS also allows designing remote laboratories thanks to the continuously released extensions that enable connection with software and hardware entities. In fact, it has been broadly used to construct such educational resources for universities all around the world. Some examples for engineering education cover several scopes like automatic control [29,30,38,39], robotics [25,40], automation [26,33], photovoltaic systems [41], supervisory systems [42]. Regarding the extensions, EJS can connect with external software widely used in academic and research laboratories, namely Java-Internet-LabVIEW (JIL) [43] and Java-Internet-Matlab (JIM) [44]. Some other tools recently released are related to robotics [40], augmented reality [45], integration and booking in Moodle [46,47], collaborative support [48], Reader Apps for Android and iOS [21], use of Javascript [49]. An emerging trend within this scope is the use of low-cost and open-source platforms such as Arduino [50], Phidget [51] or Raspberry Pi [52]. These devices provide easy-to-use hardware and software environments to develop different implementations in the fields of data acquisition, automation and engineering in general. Open-source code is a critical benefit. This feature implies that there is an increasing amount of shared information available all around the world about academic, scientific and non-professional projects. Some examples of recent applications based on these devices are now discussed. Real-time monitoring of fuel cells using LabVIEW [53] and Arduino is reported in [54,55]. Applications in solar energy facilities are presented in [56,57]. Developments related to IoT are described in [58,59]. Utilization to control in robotic applications can be found in [60] (humanoid robot) and [61] (mobile robot). They are also used for data transmission through ZigBee protocol building Wireless Sensor Networks (WSNs) [62,63,64,65,66,67]. For educational purposes, in recent years some approaches have taken advantage of these platforms. For instance, a hardware-in-the-loop scheme with educational orientation is proposed in [68]. Within the remotely accessible labs, integration of EJS with Raspberry Pi and Arduino is reported in [69,70,71].

As it has been previously mentioned, networked sensors and actuators is an active field of research concerning issues related to security [7], smart devices development [66,72], data storage and processing [35,73], architectures [74,75,76,77], software applications [58]. A noteworthy research trend is the standardization of interfaces for smart network transducers according to the IEEE 1451.0 standard [78]. This protocol aims at simplifying the connectivity of smart sensors and actuators to the communication networks. Namely, it has proven to be a useful framework to develop remote educational labs [79,80,81,82].

This paper presents a set of open-source software tools, specifically designed to cover the different issues concerning the access to sensors and actuators, which have been integrated in the authoring tool EJS. Furthermore, low-cost hardware architectures are proposed to operate with the abovementioned software tools allowing an integrated and easy access to local or remote sensors and actuators. Likewise, real application cases entirely developed with these tools, without use of commercial software, are presented.

The outline of the remainder of the paper is as follows: Section 2 gives an overview of the main features of the system. Section 3 deals with the proposal of hardware architectures and software approach for the convergence subsystem. In Section 4, a solution for unifying the communications is highlighted. Demonstrative results are presented in Section 5. Finally, the main conclusions of the work and future guidelines are addressed in Section 6.

## 2. System Overview

The design of a complete system that controls a process (plant) from the network should include two main parts or subsystems. On the one hand, a subsystem which allows sensors and actuators to be accessed from a remote network location. This subsystem must integrate sensors and actuators into the network, usually using the TCP/IP protocol, and is called *convergence subsystem* in this paper. The convergence subsystem is also a requirement to make accessible any other device—like cameras, measurement equipment, control system, etc.—of the remote plant from the network.

On the other hand, in order to make the plant accessible from the network, a Human-Machine Interface (HMI) that allows the interaction between the user and the process is necessary. This interaction is carried out through the corresponding control elements available on the HMI. It also allows the user to track the process evolution through the corresponding flow of information (video streams, parameter evolution graphics, position of an actuator, etc.). The HMI, running on the user’s computer—PC, tablet, etc.—must have network access. Some of the requirements imposed on every HMI for proper working are: rich and informative visual resources, a high level of interactivity, easy-to-use, friendly and intuitive user interface, flexibility to accept changes and so on. Also, there are various desirable features such as connectivity with other software packages, rapid design, low computational requirements and easy management of data.

**Figure 1:**
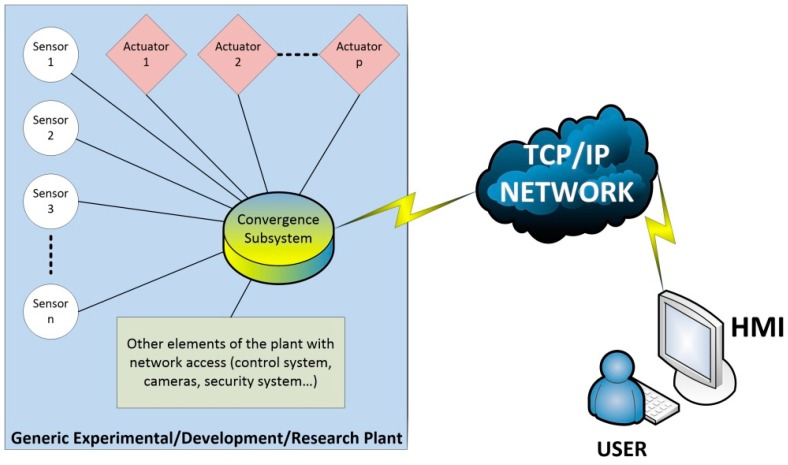
Simplified block diagram of a remotely accessed plant.

Therefore, the HMI and the convergence subsystem have to be implemented to achieve the remote use of a plant of any type—experimental, development or research plant. Figure 1 shows the complete diagram that integrates the convergence subsystem, the HMI and the rest of the parts involved in a remote access to a plant.

In addition to proprietary solutions provided by the manufacturers of some of the devices in a plant sensors, valves, motors, measurements devices, etc., there are other solutions that enable the integration in a network of any of these devices. Indeed, there are different applications to develop the convergence subsystem and the HMI. Among the most used in the scientific field (both educational and research-development), LabVIEW and MATLAB/Simulink [83] are very common. LabVIEW provides particular solutions to develop convergence subsystems for processes of any nature (electric, electronics, hydraulic, energy, etc.). MALTLAB/Simulink also offers hardware support for receiving and sending data directly from MATLAB/Simulink to lab instruments, data acquisition systems, image and video acquisition modules. Both products, LabVIEW and MATLAB/Simulink, are suitable to implement the HMI and the convergence subsystem in any type of process.

Another option relies on developing the HMI using general purpose languages like Java, C, Python, etc. This case requires advanced expertise and programming skills for designing the communication interfaces with devices, data formatting and transmission, and visualization. These factors may constitute an extra effort when the purpose is to develop and deploy the networked system in a rapid and effective manner.

However, the aforementioned commercial packages do not provide a general methodology for developing the convergence subsystem and a great economic effort is required to use them to make a process accessible from the network.

As an alternative to LabVIEW and MATLAB/Simulink, there is an interesting free authoring tool called Easy Java/Javascript Simulations (EJS). Its validity for implementing user interfaces has been widely contrasted with processes of different nature in the educational field. In fact, EJS successfully fulfills the above mentioned HMI requirements. However, EJS has not been used to implement convergence subsystems so far, since it did not include the necessary tools to connect with hardware devices. Thus, until now, the access to hardware devices from EJS was done using connections to applications such as those mentioned above. For example, in [29] the authors shows the design of a remote plant using MATLAB, LabVIEW and EJS. In [44] a networked control lab is designed using EJS and MATLAB/Simulink. A fuzzy controller is applied by a Programmable Logic Controller (PLC) using EJS to implement the remote HMI whereas a LabVIEW application communicates such interface with the PLC via Object-Linking and Embedding for Process Control (OPC) in [33].

**Figure 2:**
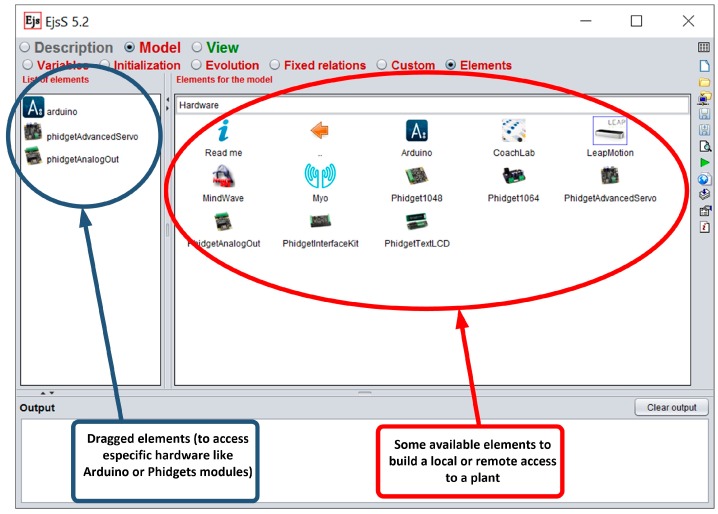
Some elements developed for EJS to access hardware platforms.

To provide EJS with the ability to connect directly to hardware devices, this paper presents a set of software tools, called *elements*. All of them are integrated in EJS. These elements provide EJS the functionality of developing convergence subsystems in a simple way, dragging and dropping them using the EJS environment, Figure 2. In addition, they solve the interaction issues between the devices connected to the convergence subsystem and the HMI—this one designed using the intuitive graphical system of EJS. All these elements are based on open software and low cost hardware platforms, and are designed by developers from different origins (universities, non-professional people) according to the open source philosophy.

## 3. The Convergence Subsystem

**Figure 3:**
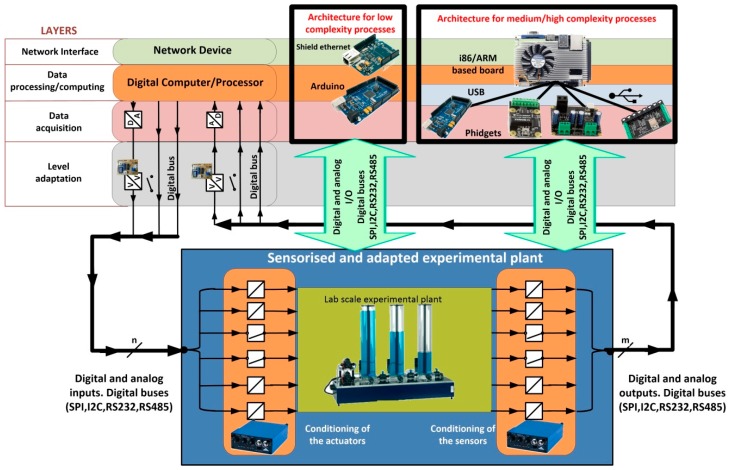
General diagram proposed to implement the convergence subsystem.

In this section, two different architectures are presented to design the hardware of the convergence subsystem. They are general architectures, depending on the complexity of the device/plant that requires being accessible from a network. The overall system is presented in Figure 3, which will explained from now on.

With respect to the plant and taken into account that it can include devices with very different nature, the plant is considered as a black box with a set of *n* input variables and *m* output variables, as can be seen at the bottom of Figure 3. The outputs are signals coming from sensors located in the plant to monitor the evolution of the process or the behavior of the plant. The inputs are signals applied to the actuators coming from a controller.

Before developing the complete convergence subsystem, the different functional layers must be established. They are required to access the plant from any TCP/IP network. The necessary layers of the proposed convergence subsystem are (top of Figure 3):

- The network interface, that is, the layer that provides access to a TCP/IP network.
- The layer of data processing/computing. It provides: TPC/IP protocol, the capability of processing to allow the establishment of network communications, and the capability of data computing. This layer also establishes the indispensable safety actions. An example of a safety action is to prevent overspeeding of an electric machine or its overheating.
- The data acquisition layer. This one provides the interface which allows the layer of data processing/computing to receive and send the information required for following the process evolution. In fact, input and output signals should be adapted according to the characteristics of the Data Acquisition System (DAQ), which should enable the processing of signals with the most common values. In addition, the DAQ usually allows digital inputs and outputs buses, such as RS232, I2C, SPI and DALLAS, among others.
- Finally, the layer of level adaptation has been introduced. Indeed, the digital and analog outputs to be processed from the plant by the DAQ need to be properly conditioned—amplification, filtering, linearization, optocoupling. This is the mission of this layer.

This structure, combined with the software tools that have been designed, has the following characteristics:

- Generality. It consists of using components with scalability and modularity. Thus, the proposed solution is adapted to plants with different data processing needs.
- Economic viability. It consists of using open hardware platforms and open source software.
- Easy design. The designer does not have to be trained on advanced knowledge in communications and electronics neither programming expertise.
- Rapid development and deployment. Due to the previous features, the time devoted to develop and implement the convergence subsystem is reduced.

The rest of this section is organized as follows: in Section 3.1 the layer of level adaptation is developed. This layer is common to both architectures proposed in this paper. The first one has been developed for processes with low complexity. It is presented in Section 3.2. The second architecture has been developed for processes with medium/high complexity and it is presented in Section 3.3. Both architectures have been included in Figure 3 (framed with black lines). Finally, in Section 3.4 the software developed to implement the convergence subsystem is presented.

### 3.1. Layer of Level Adaptation

This layer carries out the voltage range adaptation between plant inputs/outputs and the data acquisition layer. Regarding digital signals, the logic levels of actuators and sensors could be different from those of the data acquisition layer. Therefore, the ranges must be adapted to appropriate levels (for example from 0/3.3 V to 0/5 V or vice versa). Bidirectional low cost devices are included to perform this adaptation. In the case of analog signals, their adaptation to the most commonly used digital levels in A/D devices (0/3.3 V or 0/5 V) is necessary.

**Figure 4:**
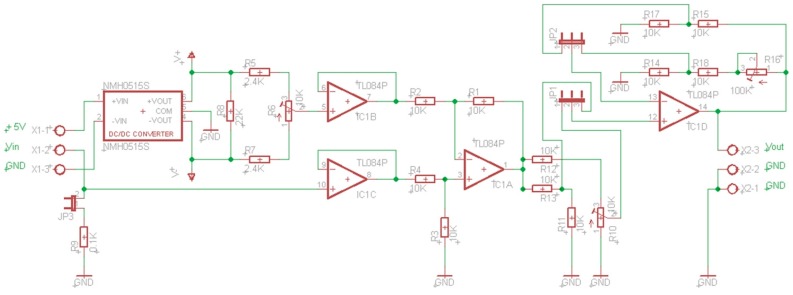
MCLAL circuit from 0/3.3 V to 0/5 V.

To carry out this levels adaptation (mapping), the designed adapter is shown in Figure 4 (Mapping Circuit of Level Adaptation Layer, MCLAL). This circuit, besides mapping voltages, converts currents to voltages. For example, the mapping of signals 4/20 mA to 0.4/2 V or 0/20 mA to 0/2 V is possible only closing the jumper JP3. Furthermore, this circuit has two advantages over the commercial devices most commonly used for this purpose. Firstly, it is only fed with 5 V versus symmetric power supply needed by many of the other. Secondly, it has independent gain setting for each channel.

**Figure 5:**
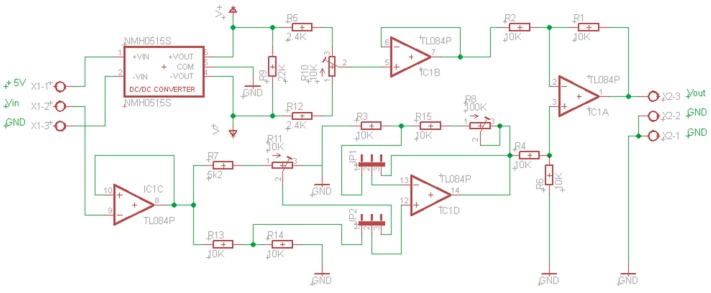
MCLAL circuit from 0/10 V to −10/10 V or, in general, to –V/+V.

**Figure 6:**
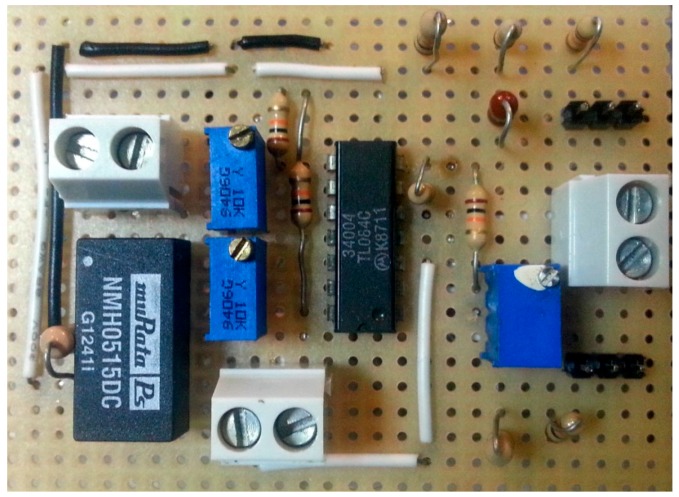
Prototype of the MCLAL circuit of Figure 5.

The layer of level adaptation should also map the output signals from the D/A converters located at the input of the plant. The actuators usually need voltages at the input of 0/10 V or −10/10 V. The output voltages from the D/A converters are usually 0/3.3 V or 0/5 V. To carry out this mapping, a circuit has been designed, Figure 5. This circuit includes, as the previous one, single voltage supply and independent setting (for negative values R10, and R11 or R8 for positive values, depending on the position of jumpers JP1 and JP2). Figure 6 shows a prototype of this circuit realized with a perfboard (DOT PCB).

Once the implementation of this layer has been solved, the next step is to deploy the remaining three layers (network interface, data processing/computing and data acquisition). Next two sections deal with the implementation of these layers for low complexity and medium/high complexity processes.

### 3.2. Architecture for Processes with Low Complexity

Processes with low complexity need simple actions of the convergence subsystem, for example, to turn the process on or to impose a certain voltage level in response to a signal generated by the user. In all these cases, the need of data processing is low. Thus, the corresponding algorithm is very simple. Also, the process security is frequently very easy to implement. Therefore a single component is proposed to complete the architecture of the remaining three layers of the convergence subsystem: a development board based on low cost microcontrollers (as can be seen at the top in the middle of Figure 3). These devices have digital inputs and outputs, analog inputs and outputs and digital serial communication buses, as well as processing power and network interfaces.

The development board chosen for this first architecture, among many others available in the market, is the free Arduino hardware platform. In recent years, Arduino has become one of the most commonly used platforms. In terms of technical specifications, Arduino development boards provide analog inputs and digital outputs. Depending on the model, the voltage ranges are 0/5 V or 0/3.3 V. They also have available serial buses with I2C, SPI and RS232 protocols. There are also cards with an Ethernet interface. The extension modules (shields) can be plugged on top of the Arduino extending its capabilities (Ethernet interface, motor control, GPS, etc.).

Arduino boards also have a large number of libraries that allow the implementation of the TCP/IP protocol and the functions of data acquisition and processing in an easy way. All these characteristics of Arduino boards make them suitable for developing the layers of data acquisition, data processing/computing and the network interface. Thus, the proposed architecture consists of an Arduino Ethernet board or a Mega board with an Ethernet shield. This architecture is an easy and cheap (under $50) solution for cases in which the need for data processing is low.

### 3.3. Architecture for Processes with Medium/High Complexity

Arduino boards allow the development of complex software. However, they neither support a high number of concurrent accesses from the network nor massive data storage/processing. Therefore, in the case of applications with these requirements, an alternative architecture is proposed for the implementation of the remaining three layers of the convergence subsystem.

In this alternative architecture, an embedded operating system has been included to manage the data storage and to increase the number of concurrent accesses from the network. In addition, it allows the use of fast development tools to design the applications needed to control the plant. This operating system must meet the characteristics listed above for the general solution. Therefore, the use of Linux, the free operating system most widely used, is proposed. The next step is to choose a hardware platform for Linux and to develop the remaining layers of the convergence subsystem.

The use of i86 and ARM based boards is proposed for this kind of processes (at the top in the right of Figure 3). The main advantages of these architectures versus others existing in the market are their adaptability and scalability. They are also the most commonly used in industrial applications. The i86 family (32 and 64 bits) has a large number of motherboards with different form factors, as for example Mini-ITX (17 × 17 cm), Nano-ITX (12 × 12 cm), Pico-ITX (10 × 7.2 cm), NUC (10 × 10 cm), etc. and a wide range of processing capabilities. From 32-bit processors up to 64 bits multi-core processors can be used, like the Intel Atom, i3, i5 and i7 processors.

In the same way, there is a high number of motherboards for the ARM processor and for industrial uses with the same form factors as the i86 boards. They can also be used to implement this second architecture. In addition, there are boards based on this processor with reduced sizes and prices, such as PhidgetSBC3 and Raspberry Pi. These boards are capable of running embedded Linux operating system and they are an intermediate solution between Arduino-based and i86/ARM boards.

All the mentioned boards have a network interface and at least one USB port. With that, all of them are suitable for developing the layers of network interface and data processing/computing. Moreover, the layer of data acquisition must be implemented to complete the structure of the convergence subsystem.

There are low cost data acquisition boards connected by USB which can be used to implement the data acquisition layer. Among all the possibilities, Phidget boards have been chosen. Several elements developed for EJS provide access to different USB cards from Phidget. This manufacturer offers a wide range of drivers for Windows, Linux and OS X operating systems for all its products. It also offers libraries for developing applications in any programming language (including Java). This last feature constitutes an advantage versus other alternatives. Phidget also provides acquisition boards with digital I/O and analog inputs, boards with digital outputs −10/10 V, PWM outputs, etc. These boards can be connected to any architecture based on i86 or ARM processors running Linux operating system. The complete system provides the processing capability necessary in the process, and allows the implementation of the data acquisition layer of the convergence subsystem. In addition, Phidget also offers a board, PhidgetSBC3 [84], based on an ARM processor with embedded Linux, which incorporates an interface with eight digital inputs, eight digital outputs, eight analog inputs and six USB ports, to which any other board needed to develop the complete structure of the convergence subsystem can be connected.

Arduino boards can be also connected to an USB port for data acquisition. To do this, the Arduino board must be programmed with the Firmata sketch, available in the integrated development environment (IDE) of Arduino. This sketch allows the communication between the computer that implements the layer of data processing/computing and the Arduino board. An element developed for EJS (see Figure 2) provides complete access to Arduino boards connected to an USB port of an i86/ARM board.

### 3.4. Software of the Convergence Subsystem

Once analyzed the different configurations of the hardware architecture, in this subsection the software to develop and implement the convergence subsystem is shown. Besides the primary functions mentioned beforehand, the convergence subsystem must also implement the three next functions:

- Communications between the convergence subsystem and the user interface.
- Control of the process behavior.
- Process safety.

The first function is responsible for the exchange of information between the user interface and the convergence subsystem. The function to control the evolution of the process is in charge of processing the information received from the plant. The safety function prevents the mishandling of the physical devices located in the laboratory.

Each process presents specific security requirements and control needs. These characteristics depend on the nature of each process and they cannot be generalized and included in a general model. However, the complexity of these two functions is the characteristic that discriminates the choice of one of the two architectures proposed for the convergence subsystem. In this sense, the first one, Arduino-based option, will only be used in cases where the safety and control requirements are very basic or non-existent. In fact, in these cases, Arduino is only necessary to provide connectivity between the user interface and the plant. For all these reasons, regarding the safety and control evolution of the plant, the only functionality proposed is a procedure of general assistance to the designer for the development of the remote plant through the application of EJS.

The i86 and ARM architectures allow the use of the Linux operating system and Java language. With the software elements designed and integrated in EJS, the user can access to the hardware devices with high-level functions. Thus, the connection to hardware devices from EJS is achieved by means of a software element for each device, represented by icons in EJS (see Figure 2). Therefore, the access to one device from EJS only consists of a drag and drop task and the configuration of the corresponding software element (port number, IP of the server, etc.).

The remote access to the plant from the network, achieved with the developed software elements/HMI, guarantees the communications with the sensors and the actuators. In addition, EJS facilitates the creation of the safety and control functions of the process evolution, because this tool provides its own mechanism for describing models of scientific and control engineering phenomena and it can be used to create the software part of a remote plant in a very simple and efficient way.

## 4. Unification of Communications Using the Industrial Modbus Protocol

In a general case, a remote plant consists of several devices that share information through a communication network, usually a fieldbus. All of these devices must be accessible remotely by other network either LAN or WAN, like the Internet. So, the communications protocols must be unified to assure interoperability between them and an effective information flow. In this regard, although the TCP/IP protocol unifies communications at transport level, the communications at application level have to be also unified. In order to tackle the last task, it is proposed the use of an open standard protocol, namely Modbus [85], which is mainly devoted to industrial communications.

**Figure 7:**
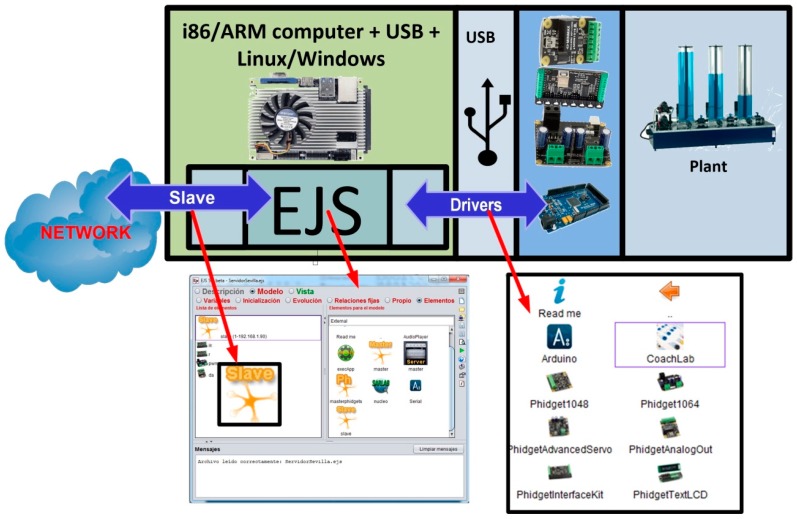
Diagram of a convergence subsystem based on i86/ARM computer, low cost data acquisition boards and EJS.

Figure 7 shows the complete hardware-software structure/architecture for processes with medium/high complexity of a convergence subsystem based on an i86/ARM computer, low cost data acquisition boards, integrated Modbus protocol and EJS like an authoring tool to develop the software needed in the convergence subsystem.

**Figure 8:**
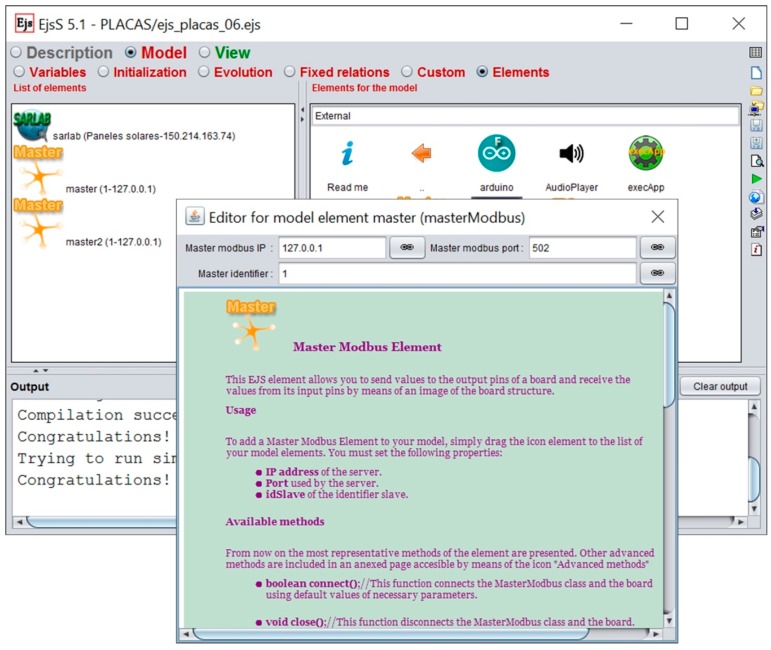
Available Elements and List of Elements added to an application in EJS.

The Modbus protocol keeps a data vector for each set of input-output pins of the data acquisition hardware. Each position inside each vector is constantly updated with the current value of the corresponding pin. Since Modbus is a master-slave protocol, the communications are initiated by the master, who is responsible for giving orders to the slaves. Slaves have only to fulfill orders received from masters. This approach requires placing the slaves on the plant side (see the lower left of Figure 7) and the master on the user interface side. Several elements have been developed in EJS to provide Modbus support both in the plant and in the remote user computer. Figure 8 shows the slave and master Modbus elements (in this case, two master Modbus elements has been added to the developed application, named master and master2). For example, to add a Master Modbus it is necessary set, in the configuration dialog box of the element, the following properties:

- IP address of the server.
- Port used by the server.
- Identifier for the slave.

These properties are introduced in a popup window that can be accessed with a double click over the Master Modbus icon, which includes a help page for the element (see Figure 8).

The use of Modbus to make a plant accessible from the network presents the following advantages:

- Within a master-slave model and in a communications unified scenario, all the elements can connect to any other element. On the contrary, the connection of all the elements is very difficult to achieve with several designers of client-server structures working independently.
- M2M (machine to machine) applications can be used to make the corresponding connections between the plant components (topological control).
- The implementation becomes easier because the communications are developed in the same way in all of the plants components.
- Different industrial components such as PLCs, digital controllers, etc., that support standard Modbus communication can be used in the plants.
- Monitoring software can be easily integrated in the system.
- There are simulators that allow the developed applications to be debugged.
- The development of the communications application is performed at a high level.

The application layer provided by Modbus solves the information exchange between the user interface/HMI and the convergence subsystem. Thus, the designer only has to implement the function calls from the master. The called functions read or write in the slave process image. Some examples of these function calls, to be included in the Initialization page of EJS, are:

- connect(): open the connection to the master. If the name of the master is, for example, myMaster, the user only needs to write myMaster.connect() to access the master. Other methods are specified in the same way.
- writeCoil(int slaveId, int index, boolean value): write a boolean value to a specific slave in index position.
- writeAnalogRegister(int SlaveId, int index, int value): write an analog value to a specific slave in index position.
- close(): close the connection to the master.
- monitorInit(): This function creates a graphical monitor to make easiest the test of all the functions calls included in the Master Modbus element.
- etc.

The designer does not have to develop the establishment, the evolution and the end of communications. This software layer hides the details of the communications technology. And so, it makes easier the development of applications when the designer has little knowledge of the development of network-based applications. For example, the inclusion of the Slave software element (in the convergence subsystem) and the Master software element (in the user interface) simply consists of dragging them from the development environment (EJS) and dropping where appropriate (Figure 8).

As mentioned, the physical implementation of the Modbus protocol has been solved with the development of two software elements in EJS: the Master element, to be included in the user interface, and the Slave element, to be included within the convergence subsystem to access each data acquisition and control board. The developed Slave element allows several concurrent connections. This ability has been exploited to implement a capability of collaborative access. Thus, once a user connects to the plant, he/she can generate a pair user/password which makes possible that other users connect to the same plant. With this collaborative capability, all the users connected to a plant can interact with the different plant devices. The collaborative actions are supervised by the Modbus software elements developed for EJS.

## 5. Results

Finally, in this section two real use cases are presented, in Section 5.1 and Section 5.2. They demonstrate the suitability of the architectures proposed in this paper to implement the convergence subsystem. The user interface/HMI, as well as the convergence subsystem, has been fully developed with EJS and the Modbus, Arduino and Phidget elements.

### 5.1. A Use Case of a Process with Low Complexity

**Figure 9:**
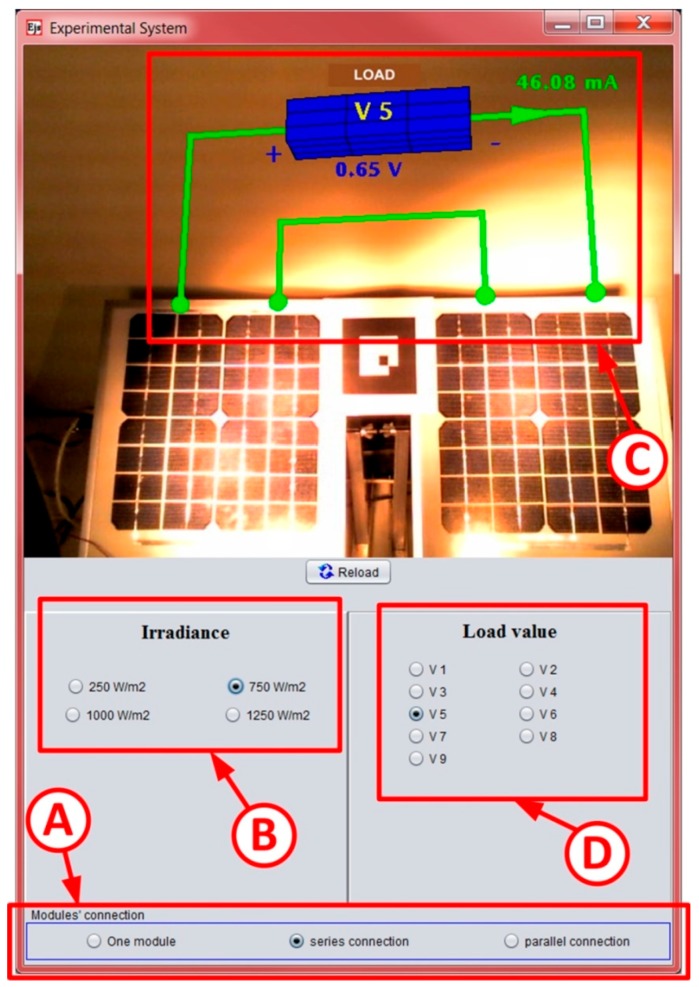
HMI designed in EJS to test the behavior of remote photovoltaic panels.

A plant to test photovoltaic panels is presented as an implementation of the architecture proposed for the processes with low complexity, Figure 9. The plant allows the obtaining of the characteristic curve of a single panel, as well as of two panels connected in series or in parallel (selectable options on the HMI, item A in Figure 9). In addition, the plant allows the variation of the radiation conditions (item B). The HMI includes an Augmented Reality system (also developed in EJS) to visualize the connections between the photovoltaic panels and the intensity and voltage of the load in real time (item C). Different loads can be selected from the HMI (item D). With this interface, the user analyzes the effects of all these parameters on the characteristic curve of the photovoltaic panels.

**Figure 10:**
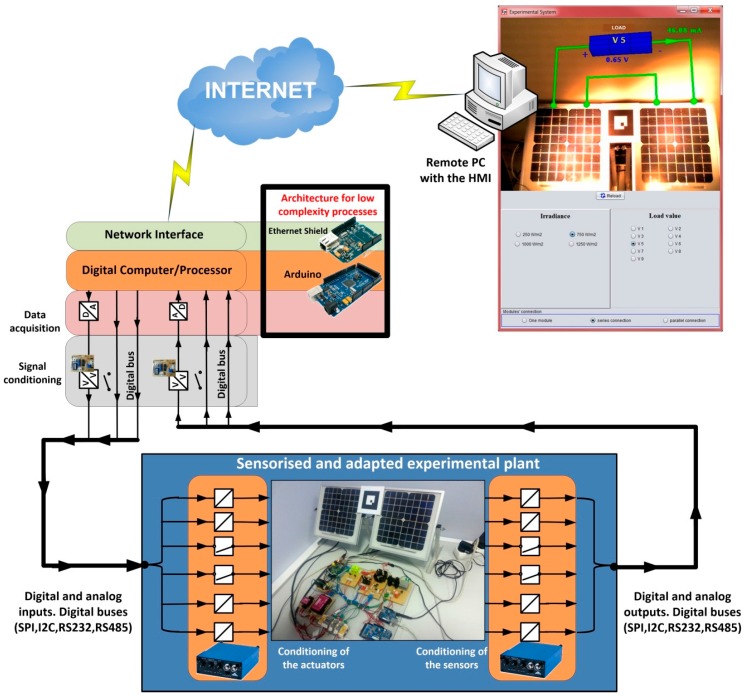
Layer structure of the convergence subsystem corresponding to the photovoltaic system.

In this case, the convergence subsystem has been developed using Arduino Mega boards, because it is not necessary an elevated processing speed on the side of the convergence subsystem. Figure 10 shows the structure of the convergence subsystem corresponding to this process, according to the layers indicated in Figure 3. As can be seen in the figure, the layers Networks Interface, Digital Computer/Processor and Data Acquisition have been implemented using two Arduino boards. An Arduino Mega board with an Ethernet Shield has been used to control the load connected to the photovoltaic panels and an Arduino Ethernet board to control the power of the luminaries which light the panels.

**Figure 11:**
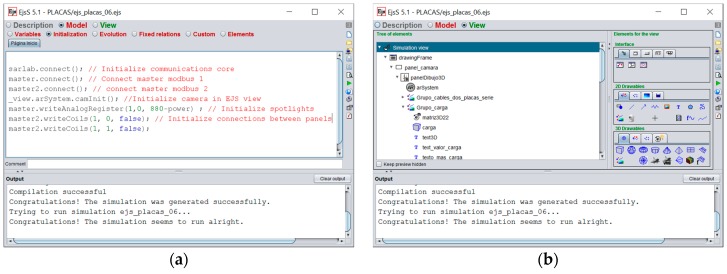
(**a**) Initialization code included in the HMI sources; (**b**) View panel in EJS with some graphical items defining the user interface.

Figure 11a shows the code introduced for the initialization in EJS: two master Modbus elements and a communications core, SARLAB. This software tool guarantees the necessary network links to communicate the user computer with the remote photovoltaic system. SARLAB is integrated in EJS like an element, and it has been developed to provide, automatically, the necessary connections to a remote system. All communications packets are encrypted by SARLAB, thus ensuring security in the information sent and received with the remote plant.

Figure 11b shows the View panel of EJS (the graphical user interface is defined by dragging and dropping graphical items—Augmented Reality system, windows, camera, texts, buttons, switches and so on. The HMI and its connection to the remote plant are designed entirely with EJS, without any other software tool.

### 5.2. A Use Case of a Process with Medium Complexity

A plant to perform tests with commercial electric machines is presented as an implementation of the architecture proposed for a processes of medium complexity, Figure 9. By means of this system, the equivalent circuit of a synchronous machine can be obtained according to the linear or Behn-Eschenburg model.

**Figure 12:**
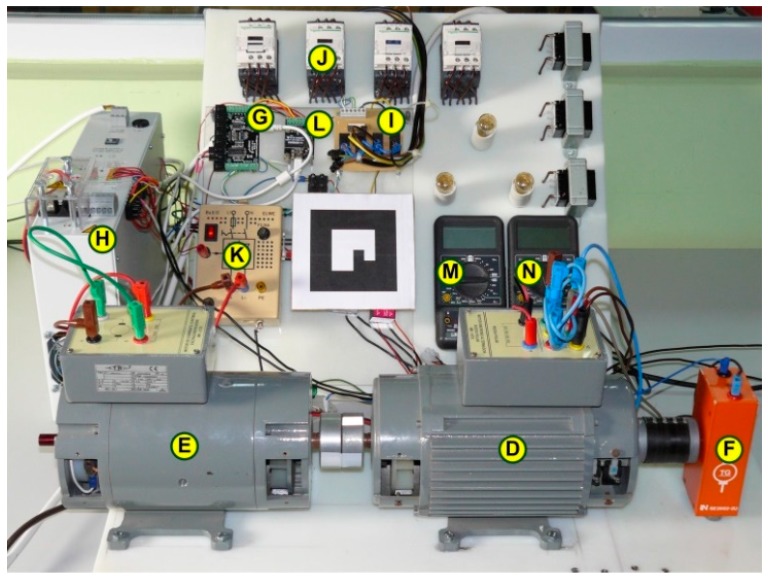
A plant to test commercial electric machines remotely.

The core of the experiment is the engine bench, which is constituted by a synchronous generator, item D in Figure 12, driven by a dc engine, item E. Both electrical machines are coupled to an analog tachometer, item F, which sends the rotational speed signal to a Phidget I/O board, item G, with analog inputs and digital outputs. This I/O board is directly connected to an USB port of the ARM processor board. This I/O board is connected to several sensors and actuators and its functions are:

- Measurement of the rotation speed.
- Measurement of the field current from the current source which supplies it, item H.
- Switching on/off current source H.
- Turning on/off the plant supply, according to the instructions received from the ARM board, through a set of optocoupled relays, item I.
- Configuring the connections suitable for each test of the synchronous generator, through a relay, item J.

The current going into the DC engine rotor is supplied by a current source, item K, which is controlled by a Phidget analog output board, item L. This device is also the controller of the field current supplied by the current source H and is connected via USB to the ARM board. Finally, a voltmeter is used to show the measure of vacuum voltage in the vacuum test, item M, and an ampermeter is used to show the short circuit current in the short circuit test, item N.

**Figure 13:**
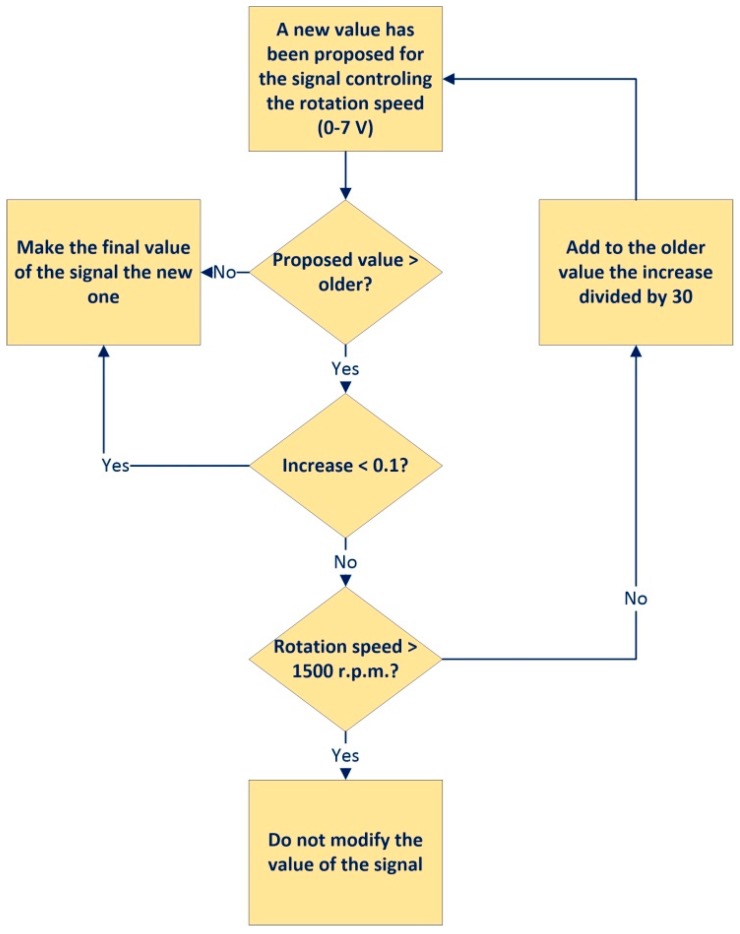
Safety algorithm for the DC engine.

In a plant with commercial electrical machines, the safety issues become drastically important. The fact of using a remote access deletes the danger for the user. However, the safety of machines goes on being necessary to consider. In order to preserve the physical elements, the convergence subsystem needs to implement the safety algorithms with a fast response time. On the one hand, several protective measures have been implemented related to the machines rotational speed. These consist basically of avoiding an abrupt increase of this parameter, whose control is available with the HMI, and stopping the rotational speed from rising above 1700 rpm either by a direct command or by an abrupt decrease of the field current. On the other hand, the second parameter that can be controlled by the user from the HMI, the field current, has been also involved in order to avoiding abrupt swings. Notice that a sudden drop in this parameter can result in a sharp rise in rotational speed. Figure 13 shows the safety control algorithm implemented to protect the remote DC engine.

**Figure 14:**
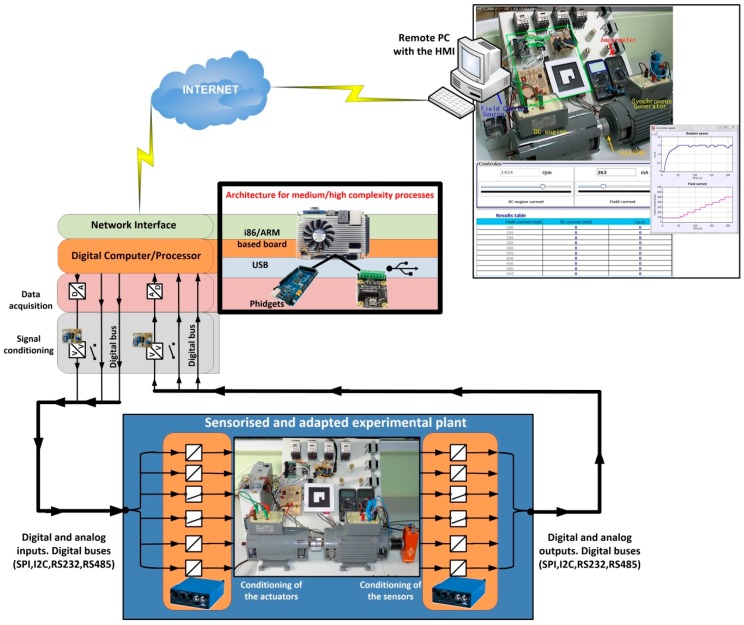
Layer structure of the convergence subsystem corresponding to the system of electric machines.

In this case, the convergence subsystem has been developed using a board based on an ARM processor. Obviously, the use of this kind of processor running EJS to implement the computer part of the convergence subsystem facilitates the development of the safety algorithms. Thereby, all the software part of the convergence subsystem is developed with EJS and the included software elements, which allow Modbus support and control of the Phidget I/O and analog boards. Figure 14 shows the structure of the convergence subsystem corresponding to this process, according to the layers indicated in Figure 3. The HMI is also shown at the top right of Figure 14.

## 6. Conclusions

In the recent years there is an increasing number of devices, plants and processes accessible from Internet. In this scenario, the implementation of two interconnected parts is necessary: the user interface and a subsystem that must integrate sensors and actuators into the network, the convergence subsystem. This paper has presented a set of tools to implement the convergence subsystem, based on a set of new capabilities included in the free authoring tool EJS for both local and remote access. The validity of EJS for implementing the user interface/HMI has already been widely proven in scientific and educational environments. With the method proposed in this paper, the two required parts (user interface and convergence subsystem) can be implemented within the same authoring tool, using only open hardware platforms and free software. In addition, a solution based on the use of the Modbus protocol for implementing the communications between those two parts through the network has been proposed in this paper. Finally, two physical architectures have been defined for developing the convergence subsystem according to the different requirements of data processing needed by the remote plant. The simplest one is based on Arduino boards, when processing needs are not high, and the most complex on devices based on i86 or ARM architectures, allowing easily implement complex algorithms to control a greater number of sensors and actuators through different data acquisition boards. To demonstrate the suitability and effectiveness of the proposed approach, two real use cases have been exposed. The tools here presented contribute to ease the development and deployment of network-based systems. Improvements to the reported work rely on the development of new elements for EJS that incorporate new functionalities, such as the use of the IEEE 1415.0 standard, support for new hardware platforms and the inclusion of PID controllers in EJS like an element, which would facilitate a control loop feedback mechanism in the experimental plant from the layer of data processing/computing.

## Abbreviations

The following abbreviations are used in this manuscript:

CPS	Cyber-Physical System
DAQ	Data Acquisition System
EJS	Easy Java/JavaScript Simulations
HMI	Human Machine Interface
I2C	Inter-Integrated Circuit
ICT	Information and Communication Technologies
IDE	Integrated Development Environment
IoT	Internet of Things
IPv6	Internet Protocol version 6
LAN	Local Area Network
M2M	Machine to Machine
MCLAL	Mapping Circuit of Level Adaptation Layer
OPC	Object-Linking and Embedding for Process Control
PLC	Programmable Logic Controller
SG	Smart Grid
SPI	Serial Peripheral Interface
TCP/IP	Transmission Control Protocol/Internet Protocol
WAN	Wide Area Network
